# Event-Preserving Neural Network Denoising for Impedance Flow Cytometry

**DOI:** 10.3390/nano16171084

**Published:** 2026-08-31

**Authors:** Leilei Shi, Charlie Jindrich

**Affiliations:** Department of Engineering, School of Engineering, Computing, and Mathematics, College of Charleston, Charleston, SC 29424, USA; jindrichca@g.cofc.edu

**Keywords:** impedance flow cytometry, neural network, single-cell analysis, signal denoising, sensing

## Abstract

Impedance flow cytometry (IFC) is a label-free technique for high-throughput electrical characterization of individual cells and other micron-scale particles based on transient impedance changes during passage through microfluidic sensing electrodes. Although lock-in demodulation and low-pass filtering are commonly used for signal conditioning, post-demodulated IFC signals can still contain residual noise, baseline drift, periodic interference, and event-like artifacts that reduce event detectability and distort quantitative features. Here, we present an event-preserving neural network-based denoising framework for post-demodulation IFC signal enhancement. The novelty of this work lies in an IFC-specific event-preserving denoising strategy that combines event-focused sampling and an event-weighted loss to suppress noise while preserving sparse bipolar particle events. A lightweight multilayer perceptron (MLP) was trained and evaluated using paired synthetic noisy and clean IFC signals generated with representative noise and artifact components, and further tested on experimental IFC measurements under challenging noise conditions. Neural network denoising improved event detection, reduced amplitude-estimation error under added white noise, and suppressed experimental baseline and background fluctuations while preserving bipolar event morphology. These results suggest that lightweight neural network denoising can serve as a practical optional enhancement step for noisy post-demodulated IFC signals, potentially supporting more reliable electrical detection and analysis of micro- and submicron-scale particles in impedance-based biosensing applications.

## 1. Introduction

Impedance flow cytometry has emerged as a powerful label-free technique for analyzing micron-scale particles, such as biological cells, bacteria, and parasites [1,2,3,4,5,6,7]. IFC characterizes particles by measuring electrical impedance changes as they traverse a microfluidic channel embedded with sensing electrodes, enabling the extraction of biophysical properties such as particle size, shape, and intrinsic electrical characteristics [8,9,10]. Owing to its label-free and non-invasive nature, IFC has attracted considerable interest for applications including drug screening [11,12], disease diagnosis [5,13], and cellular analysis [14,15]. From a broader perspective, IFC signals have been extensively studied in terms of device physics, signal acquisition strategies, and feature-level interpretation [4,16,17]. Early work established lock-in amplification and quadrature demodulation as effective means of recovering particle-induced impedance variations from high-frequency carrier signals, followed by classical digital signal-processing methods for event detection and feature extraction [9,18]. Subsequent studies have primarily focused on extracting biophysical parameters, such as size, velocity, and dielectric properties, from demodulated impedance waveforms using analytical models, handcrafted features, or learning-based inference models [19,20,21,22].

Although the specific implementation may vary depending on system design, a typical IFC signal-processing workflow can be broadly described as comprising signal acquisition, signal conditioning, event detection or segmentation, feature extraction, and downstream feature processing [4]. In most IFC systems, noise suppression is primarily performed during signal acquisition and demodulation through lock-in amplification, quadrature demodulation, and low-pass filtering, which remove carrier-frequency components and out-of-band noise. After demodulation, downstream event detection and feature extraction are often performed directly on the demodulated waveform, assuming that the remaining signal quality and baseline stability are sufficient for reliable analysis. When additional post-demodulation denoising is applied, conventional approaches such as moving-average and Savitzky–Golay (S–G) filtering are commonly used because they are computationally efficient and straightforward to implement, while wavelet-based denoising provides a more flexible multiscale approach for suppressing noise [8,10,15,23]. Their performance depends on method-specific parameter choices. For example, the degree of smoothing provided by a moving-average filter depends on the averaging-window length, while S–G filtering depends on both polynomial order and filter length. Wavelet denoising similarly depends on choices such as the wavelet basis, decomposition level, and thresholding strategy. As previously demonstrated for impedance microfluidic cytometry, stronger smoothing can improve noise reduction but may attenuate transient event amplitudes, whereas parameter choices that better preserve peak values may provide less effective noise suppression [8]. Consequently, an optimal parameter combination can be difficult to define and may vary with signal characteristics and experimental conditions. In addition, these methods are generally event-agnostic: the same smoothing operation is applied to baseline regions and particle-event regions without explicitly considering the sparse and transient nature of IFC signals.

This parameter-dependent tradeoff becomes particularly problematic for post-demodulated IFC signals because residual disturbances are not limited to simple random noise, while true particle events are short, sparse, and morphology-dependent. These disturbances can include in-band electronic noise, low-frequency baseline drift, colored noise, periodic ripple, sudden baseline shifts, and short event-like artifacts arising from flow instability, air bubbles, debris, or transient electrode disturbances. Aggressive smoothing may therefore reduce apparent noise levels but also attenuate weak events, broaden narrow transits, shift local extrema, or distort peak-to-peak transit timing. Thus, an effective post-demodulation enhancement method should suppress residual disturbances while preserving the transient bipolar morphology needed for reliable event detection and quantitative feature extraction. These challenges motivate the development of structure-aware signal enhancement methods that can distinguish localized particle-event morphology from background fluctuation. In particular, an event-preserving denoising strategy should reduce residual post-demodulation noise while retaining the positive and negative lobes, event amplitude, and timing information required for downstream IFC analysis.

Neural networks have demonstrated strong performance in signal denoising tasks across a wide range of scientific and engineering applications. Learning-based denoising approaches have been successfully applied to seismic signals, GNSS time series, biomedical recordings such as electrocardiograms, electroencephalograms, nanopore-based electrical sensing, and other measurement signals contaminated by complex noise sources [24,25,26,27]. In these applications, neural networks can learn nonlinear mappings between noisy and clean signals, enabling the suppression of structured noise, baseline disturbances, and artifacts while preserving subtle signal characteristics that are often degraded by conventional filtering methods. These successes suggest that neural-network-based denoising may provide a promising strategy for enhancing post-demodulation IFC waveforms. Although machine learning has attracted increasing attention in IFC, most existing studies have focused on classification, parameter estimation, or feature extraction rather than signal denoising [19,28,29]. For example, Honrado et al. developed an RNN-based framework for real-time inference of particle size, velocity, and position from IFC measurements [19], while LSTM-based models have been employed for multiparametric signal templating and leukocyte differential analysis [29]. These studies demonstrate the potential of neural networks for extracting information from IFC signals, but they do not address the problem of improving signal quality prior to event detection and feature extraction.

In this study, we introduce an event-preserving neural network framework for post-demodulation IFC signal denoising. The proposed method is designed as an optional signal-processing step after conventional demodulation and before event detection and feature extraction. Its primary objective is to improve signal quality while preserving transient event morphology, thereby supporting more reliable downstream feature extraction rather than directly performing particle classification. Unlike conventional smoothing methods, the proposed framework is designed specifically around the sparse bipolar morphology of IFC particle events. By combining event-focused sampling with an event-weighted loss, the method emphasizes preservation of transient particle signatures while suppressing residual noise, baseline fluctuation, periodic interference, and event-like artifacts. Its significance lies in providing a lightweight, hardware-independent post-demodulation enhancement step that can improve event detectability and support more reliable downstream feature extraction.

The network is trained using paired synthetic IFC signals, in which representative noisy waveforms are generated by adding representative noise and artifact components to clean bipolar particle-event signals. These paired signals provide known clean targets for supervised training and quantitative evaluation. After training, the model maps noisy signal windows to clean output windows, providing a denoised waveform for downstream event detection and feature extraction. The performance of the proposed framework is evaluated using synthetic IFC datasets with known ground truth and experimentally acquired IFC signals under challenging noise conditions. We compare raw demodulated signals and artificial neural network (ANN)-denoised signals in terms of event-detection performance, threshold sensitivity, noise robustness, feature consistency, and experimental signal quality. The results show that the proposed event-preserving ANN improves true-event detection, particularly for weak or noisy particle transits, while maintaining comparable event morphology for commonly detected events. This work demonstrates that lightweight neural denoising can serve as a practical post-demodulation signal enhancement strategy for IFC, bridging the gap between conventional lock-in signal conditioning and reliable downstream event-level analysis.

## 2. Materials and Methods

### 2.1. Overall Workflow

**Figure 1:**
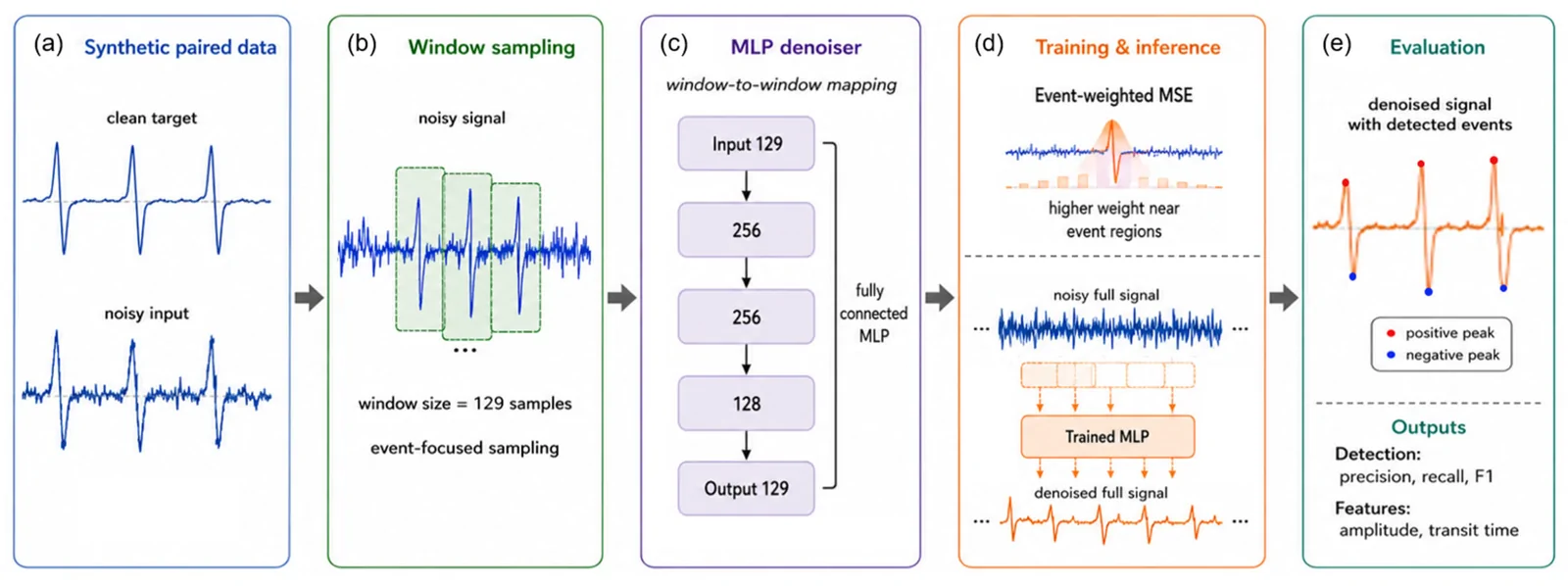
Event-preserving ANN denoising framework for IFC signals. (**a**) Paired synthetic IFC signals are generated by combining clean bipolar particle-event waveforms with representative noise and artifacts. (**b**) Short overlapping windows are sampled from the noisy signal, with event-focused sampling used to emphasize particle transits. (**c**) A lightweight MLP maps noisy input windows to clean output windows. (**d**) The network is trained using an event-weighted mean-squared-error loss and then applied across the full noisy signal during inference to reconstruct the denoised waveform. (**e**) The denoised signal is subsequently used for event detection and quantitative feature extraction, including peak amplitude and transit time. The ellipses indicate signal continuation, and the dashed boxes represent the sampled signal windows.

The proposed workflow was developed as a post-demodulation signal enhancement strategy for impedance flow cytometry, as summarized in Figure 1. The workflow begins with paired synthetic data generation, where clean bipolar IFC particle-event signals are combined with representative noise and artifact components to produce corresponding noisy input signals (Figure 1a). The clean waveform serves as the target signal, while the noisy waveform serves as the ANN input. This paired design enables supervised learning, allowing the model to learn a direct mapping from noisy post-demodulation waveforms to clean event-preserving signals. The noisy signal is then divided into short overlapping windows for training (Figure 1b). Each training sample consists of a 129-sample noisy input window and the corresponding 129-sample clean target window. Because IFC events are sparse and most time points correspond to baseline, event-focused sampling is used to increase the number of training windows centered near true particle events. Baseline windows are also included, but the sampling strategy intentionally emphasizes particle transits so that the model learns to preserve short bipolar events rather than simply reconstructing the background.

A lightweight fully connected MLP is then trained to perform window-to-window denoising (Figure 1c). The network maps each noisy input window to a clean output window of the same length. The MLP architecture consists of an input layer with 129 nodes, three hidden layers with 256, 256, and 128 nodes, and an output layer with 129 nodes. Rectified linear unit activation functions are used between fully connected layers. This compact architecture is suitable for IFC denoising because particle transits are localized waveform features with limited long-range inter-event dependency. The MLP is trained using an event-weighted mean-squared-error loss and then applied to full-length noisy signals by sliding-window inference (Figure 1d). During training, samples near true particle events are assigned higher weights in the loss function, encouraging the network to accurately reconstruct the positive and negative lobes of the bipolar waveform. During inference, the trained MLP is moved across the full noisy signal. Since adjacent windows overlap, each time point is predicted multiple times, and the final denoised signal is reconstructed by averaging the overlapping output windows. This procedure produces a continuous denoised IFC waveform while reducing window-boundary artifacts.

Finally, the denoised signal is passed to the downstream event detection and feature extraction pipeline (Figure 1e). Events are detected using an amplitude-threshold method applied to the median-corrected signal. Detection performance is evaluated using precision, recall, and F1 score by matching detected events to ground-truth event centers. Event-level features, including peak amplitude and transit time, are extracted from local event windows and compared with the corresponding features obtained from raw noisy signals. In this way, the workflow directly tests whether ANN-based post-demodulation denoising improves event detectability while preserving the waveform morphology needed for quantitative IFC analysis. Synthetic signal generation, event detection, and quantitative analysis were implemented in Python 3.11.11 (Python Software Foundation, Wilmington, DE, USA). ANN training and inference were performed using PyTorch 2.5.1 (PyTorch Foundation, San Francisco, CA, USA).

### 2.2. Synthetic IFC Signal Generation

Synthetic IFC signals were generated to provide paired noisy-clean training data with precise ground-truth event annotations. To support controlled training and evaluation, the synthetic framework was designed to span representative post-demodulation disturbances rather than reproduce a single experimental noise condition exactly. This approach provides known clean ground truth while enabling systematic evaluation of denoising performance across variable noise conditions. The generation framework models the complete signal chain from particle-transit physics through lock-in amplifier demodulation, incorporating representative noise sources and measurement artifacts encountered in experimental IFC recordings.

**Figure 2:**
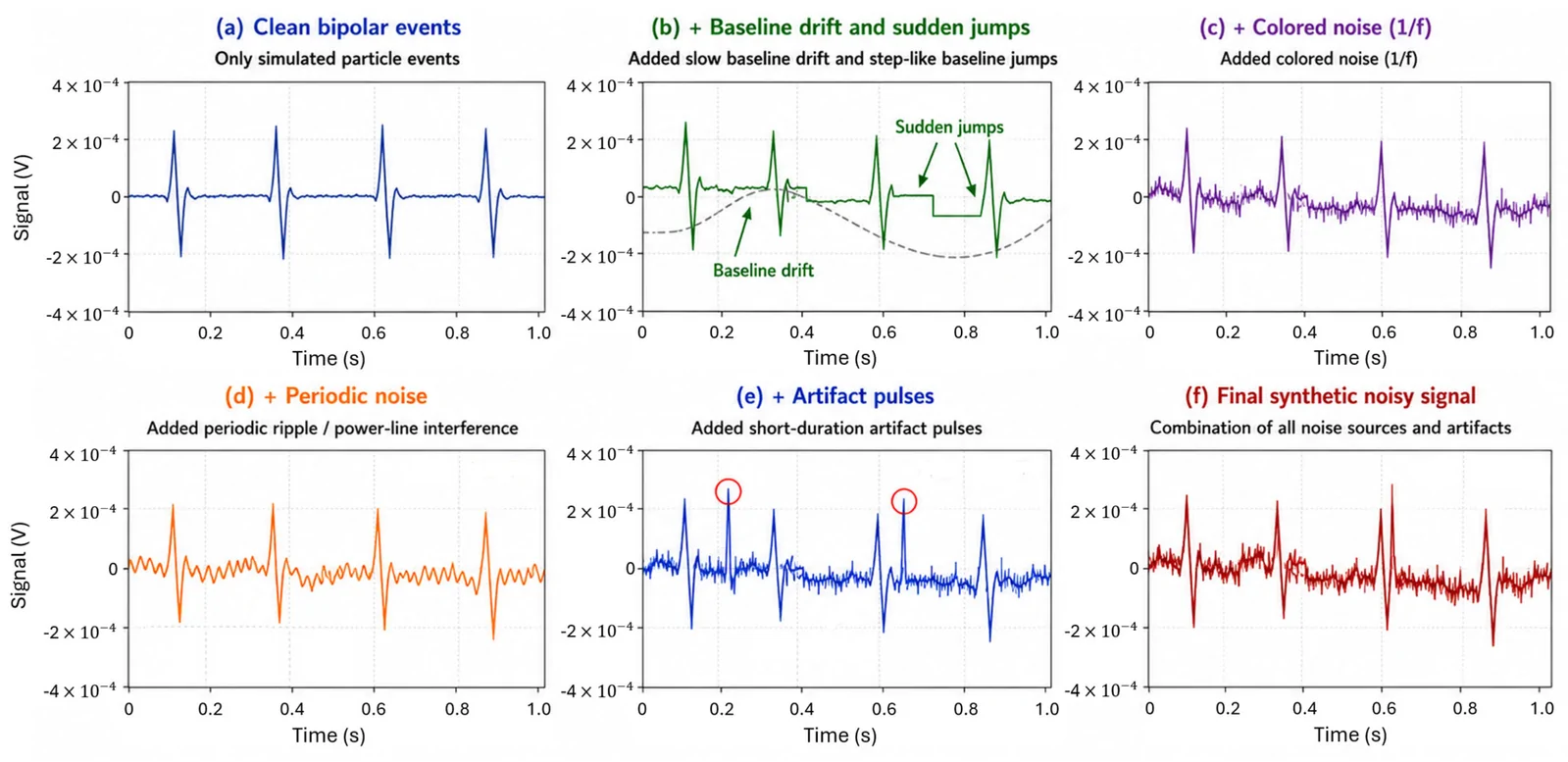
Synthetic IFC signal generation with representative noise and artifacts. (**a**) Clean bipolar particle-event waveforms were generated as ground-truth target signals. Representative non-ideal components were then added, including (**b**) baseline drift and sudden baseline jumps, (**c**) colored residual noise, (**d**) periodic ripple, and (**e**) short event-like artifact pulses (indicated by red circles). (**f**) The final noisy synthetic signal was formed by combining the clean event signal with all disturbance components and was used as the noisy input for supervised ANN denoising.

As shown in Figure 2, the total raw signal $S_{raw}(t)$ was modeled as the linear superposition of clean particle events and multiple independent noise and artifact components:

(1) $$S_{raw}\left(t\right)=S_{clean}\left(t\right)+\eta_{white}\left(t\right)+\eta_{colored}\left(t\right)+D\left(t\right)+P\left(t\right)+J\left(t\right)+B\left(t\right)+A\left(t\right)$$

where $S_{clean}(t)$ represents the ideal particle impedance response, $\eta_{white}(t)$ and $\eta_{colored}(t)$ are white and colored noise components, D(t) is slow baseline drift, P(t) is periodic interference, J(t) represents sudden baseline jumps, B(t) models channel blockage artifacts, and A(t) captures spurious artifact pulses.

Individual particle-transit events were modeled as asymmetric bipolar Gaussian pulses that capture the characteristic positive-negative impedance signature observed when a particle passes through the microfluidic interrogation zone [30]:

(2) $$p\left(t\right)=A\left[\exp\left(-\frac{{\left(t-t_{c}+\sigma \right)}^{2}}{2\sigma^{2}}\right)-\exp\left(-\frac{{\left(t-t_{c}-\sigma \right)}^{2}}{2(\alpha \sigma {)}^{2}}\right)\right]$$

where $t_{c}$ is the event center time, A is the pulse amplitude, σ is the characteristic width, and α is an asymmetry factor controlling the relative width of the negative lobe. The pulse amplitude scaled with the cube of particle diameter ($A\propto d^{3}$), reflecting the volumetric dependence of impedance in the resistive pulse sensing regime. Particle diameters were drawn from $N(d_{nom},(CV\cdot d_{nom}{)}^{2})$ with CV=0.15 for nominal diameters $d_{nom}$ of 3, 4, or 5 μm. Trajectory-dependent amplitude variations were incorporated by multiplying each amplitude by a random factor from $N(1.0,0.2^{2})$, while pulse widths were drawn from $N(350\mu s,{(70\mu s)}^{2})$ and asymmetry parameters from U(0.7,1.5). Event counts followed Poisson statistics with expectation $N_{events}=C\cdot Q\cdot T$, where C is particle concentration, Q is flow rate, and T is acquisition duration.

The disturbance models in Equation (1) were selected to represent non-ideal signal components commonly encountered in impedance-based microfluidic measurements [4,31,32]. Their specific amplitudes, frequencies, occurrence rates, and time constants were chosen to produce disturbances comparable in scale to the particle-event signals and background variations observed in our experimental IFC recordings, while spanning conditions from moderate background fluctuation to severe transient interference. These values were therefore intended to define representative stress-test conditions rather than universal IFC noise parameters. For the present simulations, white noise was modeled as $\eta_{white}(t)∼N(0,(2\times 10^{-4}{)}^{2})$, corresponding to a standard deviation of $2\times 10^{-4}$. Colored noise with a $1/f^{\alpha }$ power spectrum was generated using α=1.0 and $\sigma =1\times 10^{-4}$. Baseline drift D(t) consisted of a 0.03 Hz sinusoidal component, a linear trend with a total drift of $1\times 10^{-4}$, and a cumulative random walk. Periodic interference P(t) included a 60 Hz component with an amplitude of $1\times 10^{-5}$, a low-frequency flow-related component at 0.5–3.0 Hz with an amplitude of $8\times 10^{-5}$, and its second harmonic with an amplitude of $3\times 10^{-5}$. Sudden baseline jumps J(t) occurred 1–4 times per recording and were modeled as step disturbances with exponential recovery, with τ∼U(2,8) s. Blockage artifacts B(t) occurred with a 30% probability, with 1–2 artifacts per affected recording, and were modeled as Gaussian deformations lasting 0.3–2.0 s. Short event-like artifact pulses A(t) occurred 2–7 times per recording and were modeled as either monophasic Gaussian or distorted bipolar pulses with amplitudes drawn from $N(0,{\left(6\times 10^{-4}\right)}^{2})$.

To emulate lock-in amplifier demodulation, both the raw composite signal and the clean event signal were processed through a cascade of four first-order Butterworth low-pass filters with zero-phase (forward-backward) implementation at a cutoff frequency of 500 Hz. The filtered clean signal served as the ground-truth target, while the difference between the filtered noisy and filtered clean signals defined the post-demodulation residual noise floor. Synthetic recordings were generated at $f_{s}=7196$ Hz with 30 s duration (215,880 samples per recording). Datasets were produced for nominal particle diameters of 3, 4, and 5 μm, each with five independent random seeds (15 unique recordings), at a flow rate of 2.0 μL/min and concentration of $10^{3}$ particles/μL. Ground-truth annotations containing event times, sample indices, diameters, amplitudes, widths, and asymmetry parameters were saved for all recordings, along with separate artifact annotations to enable rigorous evaluation of event-detection performance.

### 2.3. Threshold-Based Detection and Noise-Robustness Analysis

Event detection was performed using an amplitude-threshold method applied to the median-corrected signal. For each raw or ANN-denoised waveform, the signal baseline was first corrected by subtracting the median value. The noise level was then estimated as the standard deviation of the median-corrected signal, and the detection threshold was defined as $T=\alpha \cdot \sigma_{noise}$, where $\sigma_{noise}$ is the standard deviation of the median-corrected signal and α is the threshold factor. Candidate events were identified as peaks in the absolute value of the median-corrected signal exceeding this threshold, with a minimum peak-distance constraint used to reduce duplicate detections from the same bipolar event. For the direct raw-versus-ANN detection comparison, a fixed threshold factor of α = 4.0 was used for both raw and ANN-denoised signals. For the threshold-sweep analysis, α was varied over a range of values, and precision, recall, and F1 score were recalculated at each threshold factor.

Noise-robustness analysis was performed to evaluate how the trained ANN denoiser responded to increasing levels of controlled additive noise. For this analysis, clean synthetic IFC signals from the held-out test set were used as the starting point, and zero-mean Gaussian white noise was added at different standard deviations. The tested noise standard deviations were $5\times 10^{-5}$ to $1\times 10^{-3}$. This controlled noise sweep was designed to isolate the effect of increasing random noise amplitude while keeping the underlying event locations and clean event waveforms fixed. For each added noise level, the noisy signals were processed by the trained ANN denoiser using the same sliding-window inference procedure described above. Event detection and feature extraction were then performed on both the raw noisy signal and the ANN-denoised signal. Detection F1 score, peak-amplitude relative error, and transit-time relative error were calculated for each condition. Unless otherwise stated, the noise-robustness analysis used a fixed threshold factor of α = 2.0, with the detection threshold recalculated separately for each signal from its standard-deviation-based noise estimate. This ensured that the threshold reflected the noise level of each raw or ANN-denoised waveform while allowing performance to be compared consistently across increasing additive-noise conditions.

### 2.4. Experimental Data Validation

To evaluate the performance of the proposed ANN-based denoising method under real experimental conditions, we utilized a single population of polystyrene beads with a nominal diameter of 7.32 μm (catalog number FSDG007, Bangs Laboratories, Inc., Fishers, IN, USA). The bead suspension was diluted to a concentration of $1\times 10^{5}$ beads per milliliter in 1× PBS buffer solution (Thermo Fisher Scientific, Waltham, MA, USA). All experiments were conducted using a microfluidic chip (Micronit, Enschede, The Netherlands) with a 30 μm × 30 μm channel cross-section, containing two pairs of facing platinum (Pt) electrodes (20 μm width, 20 μm spacing). The bead suspension was driven through the channel at a flow rate of 0.5 μL/min using a flow rate controller (Fluigent, Fontainebleau, France).

Signal detection was performed using an HF2LI lock-in amplifier coupled with an HF2TA transimpedance amplifier module (Zurich Instruments, Technopark, Zurich, Switzerland). A 1 MHz alternating current (AC) excitation signal with a peak amplitude of 0.8 V was applied across the electrode pairs to induce impedance changes during particle transit. The resulting signal was demodulated using a fourth-order low-pass filter with a 460 Hz bandwidth to extract the magnitude envelope. Data were digitized at a sampling rate of 7196 Sa/s, and subsequently processed using the Python-based workflow described above.

The acquired experimental signals were used directly as input to the trained ANN model without any preprocessing beyond what is inherent to the lock-in amplifier output. The denoised output was evaluated using the same event-detection pipeline applied to the synthetic data, and the resulting detection performance and waveform fidelity were compared against the raw lock-in amplifier output.

## 3. Results and Discussion

### 3.1. ANN Denoising Preserves Bipolar IFC Event Morphology

**Figure 3:**
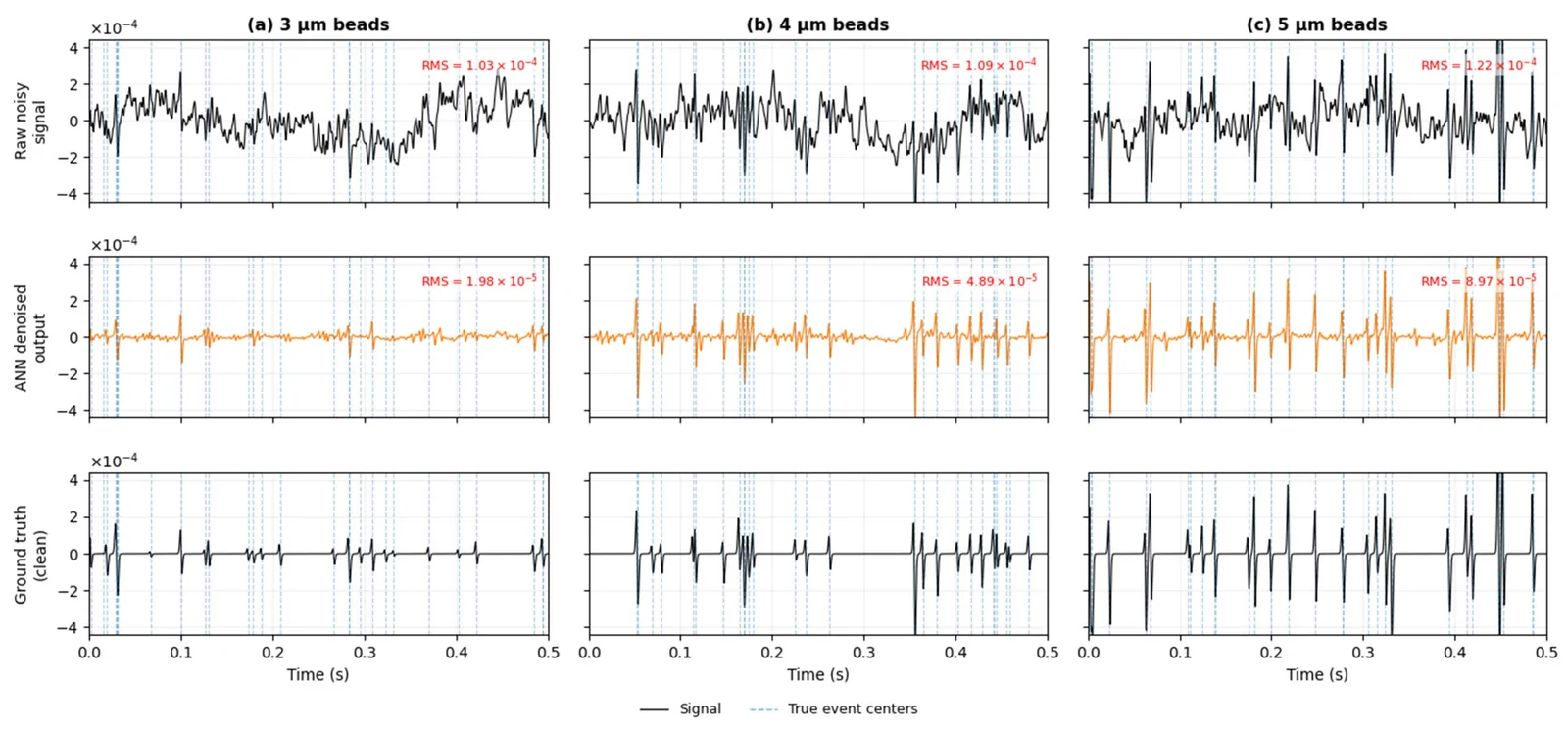
Representative ANN denoising results for synthetic IFC signals. (**a**–**c**) Raw noisy inputs, ANN-denoised outputs, and ground-truth clean signals are shown for simulated 3, 4, and 5 µm beads. Blue dashed lines indicate true-event centers.

The trained ANN was first evaluated on synthetic IFC signals with known ground-truth event locations and clean target waveforms. This controlled synthetic framework enables direct assessment of denoising performance while allowing the disturbance conditions to be systematically varied. Accordingly, rather than reproducing a single experimental waveform, the dataset was designed to span representative disturbance types and amplitudes observed across different devices, flow conditions, electronics, and acquisition settings. Representative denoising results for simulated 3, 4, and 5 µm beads are shown in Figure 3. For each particle size, the raw noisy input signal, ANN-denoised output, and corresponding ground-truth clean signal are displayed over the same 0.5 s window. The blue dashed lines indicate the true-event centers, allowing direct visual comparison between the reconstructed waveform and the underlying ground truth. The raw noisy signals exhibited substantial baseline fluctuation and residual in-band noise, which made some particle events difficult to distinguish from the background. This effect was especially evident for smaller 3 µm beads, where the event amplitudes were lower and therefore more easily masked by noise. After ANN denoising, the background fluctuation was strongly reduced, and the sparse bipolar event signatures became more distinguishable. Importantly, the denoised signals retained the localized positive and negative lobes associated with particle transits, rather than being converted into broad or distorted peaks. Across all three particle sizes, the ANN-denoised outputs followed the temporal distribution of the ground-truth events and preserved the sparse event structure of the clean signals. The reduction in signal fluctuation is also reflected in the RMS values shown in Figure 3, which decreased from $1.03\times 10^{-4}$ to $1.98\times 10^{-5}$ for 3 µm beads, from $1.09\times 10^{-4}$ to $4.89\times 10^{-5}$ for 4 µm beads, and from $1.22\times 10^{-4}$ to $8.97\times 10^{-5}$ for 5 µm beads after ANN denoising.

**Figure 4:**
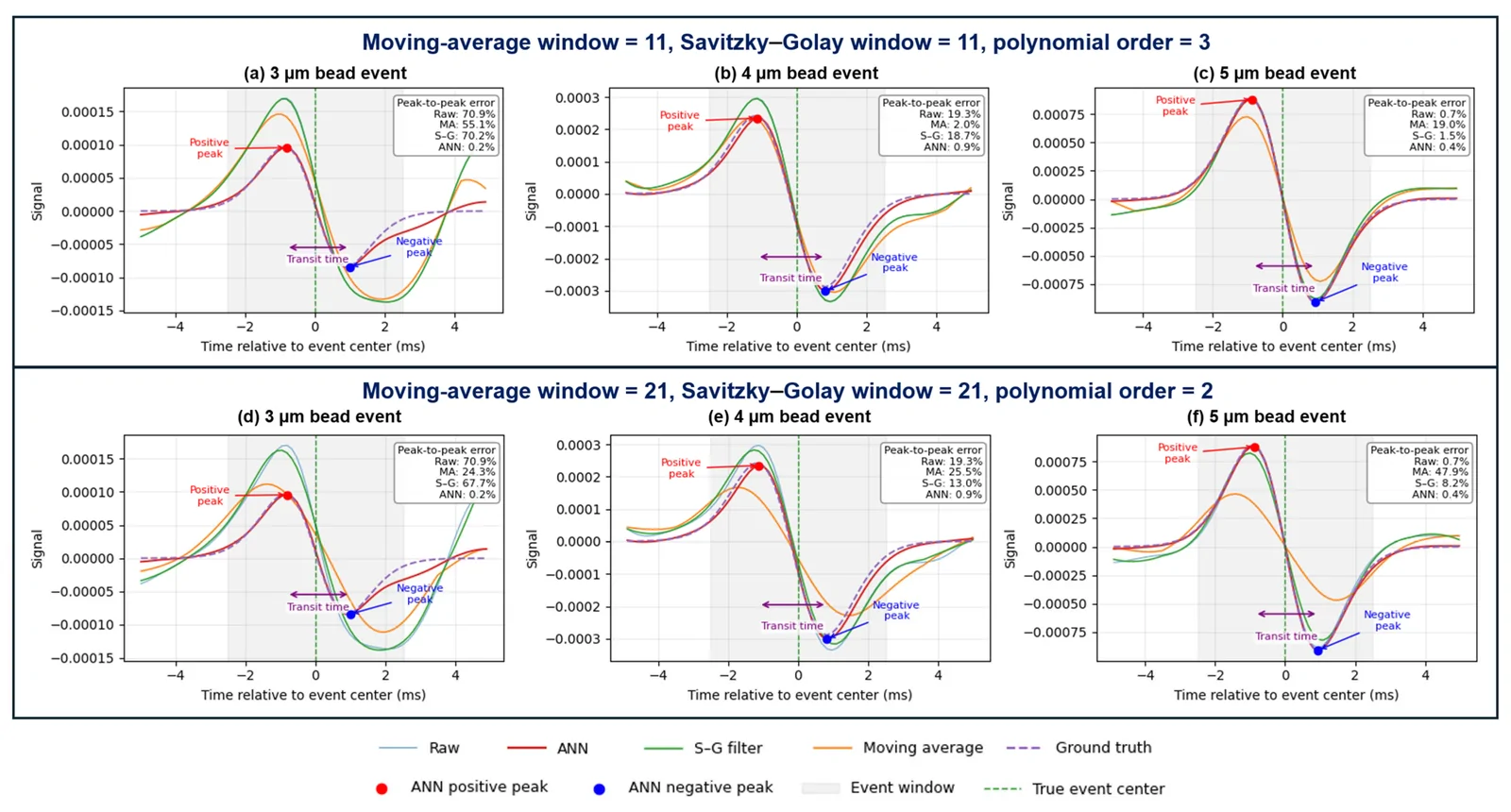
Comparison of event morphology preservation using conventional filtering and ANN denoising under different filter parameters. Representative 3, 4, and 5 µm bead events are compared using the raw noisy signal, moving-average filtering, Savitzky–Golay (S–G) filtering, ANN denoising, and the ground-truth clean waveform. (**a**–**c**) Moving-average and S–G window lengths of 11 samples were used, with a polynomial order of 3 for the S–G filter. (**d**–**f**) Stronger smoothing was applied using window lengths of 21 samples and an S–G polynomial order of 2.

To further evaluate event morphology at higher temporal resolution, one event was randomly selected from each particle-size group and compared with conventional moving-average and S–G filtering (Figure 4). The comparison is intended to illustrate the tradeoff between smoothing strength and event preservation rather than to provide an exhaustive benchmark of all possible denoising approaches. Two representative parameter settings were therefore examined to assess how conventional smoothing performance depends on filter configuration. With a window length of 11 samples and an S–G polynomial order of 3 (Figure 4a–c), the ANN produced peak-to-peak errors of only 0.2%, 0.9%, and 0.4% for the representative 3, 4, and 5 µm events, respectively. In comparison, the corresponding moving-average errors were 55.1%, 2.0%, and 19.0%, while the S–G errors were 70.2%, 18.7%, and 1.5%. Thus, although the conventional methods preserved the overall bipolar morphology of the larger events under some conditions, their performance varied substantially with event amplitude and filter configuration, and substantial error remained for the weaker 3 µm event. When stronger smoothing was applied using a window length of 21 samples and an S–G polynomial order of 2 (Figure 4d–f), the moving-average peak-to-peak errors changed to 24.3%, 25.5%, and 47.9% for the 3, 4, and 5 µm events, respectively, while the corresponding S–G errors were 67.7%, 13.0%, and 8.2%. In contrast, the ANN errors remained 0.2%, 0.9%, and 0.4%, respectively. This result illustrates the tradeoff between noise suppression and event preservation that arises from parameter selection in conventional smoothing methods, consistent with previous observations that increasing the S–G filter length can improve noise reduction while simultaneously reducing signal peak amplitude [8].

In comparison, the ANN-denoised traces remained closely aligned with the ground-truth bipolar waveform for the representative 3, 4, and 5 µm events. Both the positive and negative lobes were preserved, and the locations of the extracted positive and negative peaks also remained consistent with the expected event morphology, supporting reliable estimation of peak-to-peak amplitude and transit time. For the representative 3, 4, and 5 µm events, the peak-to-peak and transit-time errors remained small, indicating that the denoising process did not substantially distort the key waveform features required for quantitative IFC analysis. These results demonstrate that the proposed ANN learned an event-preserving denoising mapping rather than simply applying generic smoothing to the signal. At the stream level, the model reduced residual noise and improved event visibility; at the individual-event level, it preserved the bipolar waveform structure needed for downstream feature extraction. This morphology preservation is critical because IFC analysis relies not only on detecting the presence of events but also on accurately measuring event amplitude, timing, and shape-related features. These representative event-level comparisons are complemented by the dataset-level quantitative analyses of detection performance and feature accuracy presented in the following sections.

### 3.2. ANN Denoising Improves Event-Detection Performance

**Figure 5:**
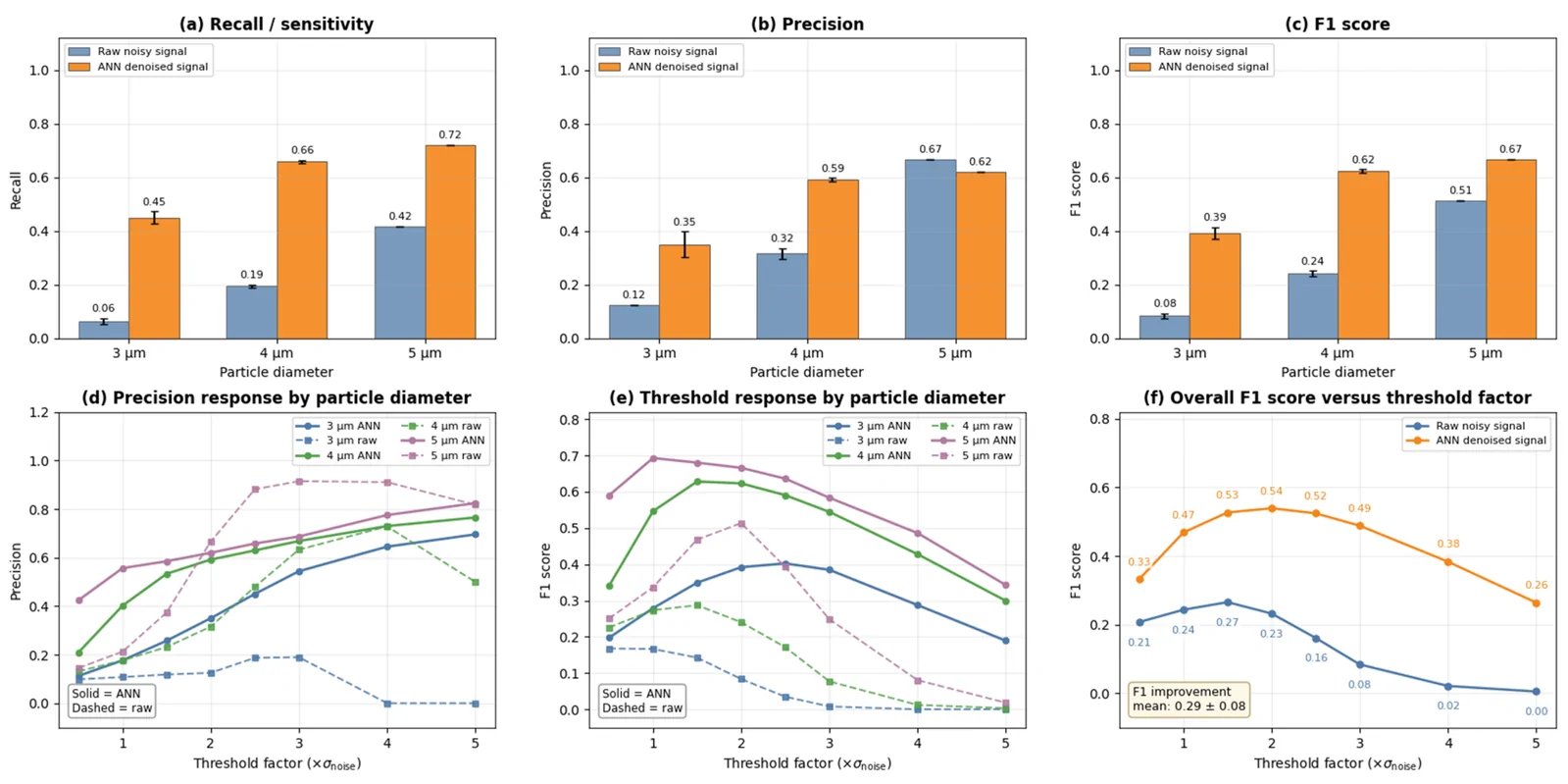
Event-detection performance and threshold sensitivity of ANN-denoised synthetic IFC signals. (**a**–**c**) Detection recall, precision, and F1 score were compared between raw noisy signals and ANN-denoised signals for simulated 3, 4, and 5 µm beads. The ANN substantially improved recall and F1 score across particle sizes, with the strongest improvement observed for smaller, lower-amplitude events. (**d**–**f**) Threshold-sweep analysis further shows that detection performance depends on the selected threshold factor, and that ANN-denoised signals maintain higher overall F1 scores over a broad threshold range.

After confirming that the ANN preserved bipolar event morphology, we evaluated whether the denoised signals improved event-detection performance. Using the amplitude-threshold detector described in Section 2.3 with a fixed threshold factor of α = 4.0, detection results for simulated 3, 4, and 5 µm beads are summarized in Figure 5a–c. Compared with raw noisy signals, the ANN-denoised signals produced substantially higher recall across all particle sizes. This improvement was especially pronounced for the smaller 3 µm beads, where the raw signal produced low recall due to the relatively weak event amplitudes. After denoising, many events that were previously obscured by baseline fluctuation and residual noise became detectable. The improvement in recall translated directly into higher F1 scores for all tested particle sizes. For 3 µm beads, the F1 score increased from 0.08 for the raw signal to 0.39 after ANN denoising. For 4 µm beads, the F1 score increased from 0.24 to 0.62. For 5 µm beads, the F1 score increased from 0.51 to 0.67. These results indicate that the ANN improved the overall balance between true-event detection and false detections. The gain was most significant for smaller particles, consistent with the expectation that low-amplitude events benefit most from noise suppression. Precision also improved for 3 and 4 µm beads after ANN denoising. For 5 µm beads, however, the ANN precision was slightly lower than the raw-signal precision at the selected threshold. This result does not necessarily indicate poorer denoising performance. Instead, it reflects the fact that ANN denoising increased the number of detected events and changed the effective signal-to-noise distribution used by the threshold-based detector. Because the same fixed thresholding strategy was applied across raw and denoised signals, the selected threshold may not be optimal for every particle size and signal condition.

To further investigate this effect, threshold-sweep analysis was performed (Figure 5d–f). The precision response by particle diameter showed that 5 µm ANN precision increased as the threshold factor was raised, indicating that the lower precision observed at the selected threshold was strongly influenced by threshold setting. Similarly, the F1-score response by particle diameter showed that ANN-denoised signals maintained higher F1 scores than raw signals across a broad range of thresholds, although the optimal threshold differed by particle size. The overall threshold sweep confirmed that the ANN-denoised signals achieved consistently higher average F1 scores than raw noisy signals, with an average F1 improvement of approximately 0.29 across the tested threshold range. These results demonstrate that event-preserving ANN denoising improves the detectability of synthetic IFC events, particularly under conditions where particle amplitudes are low relative to the residual noise background. The threshold-sweep analysis also highlights an important practical consideration: denoising changes the signal statistics, so the optimal event-detection threshold may shift after ANN processing. Therefore, downstream threshold selection should be evaluated jointly with the denoising method rather than assumed to be identical for raw and denoised signals.

### 3.3. Noise Robustness and Detection-Set Bias Analysis

**Figure 6:**
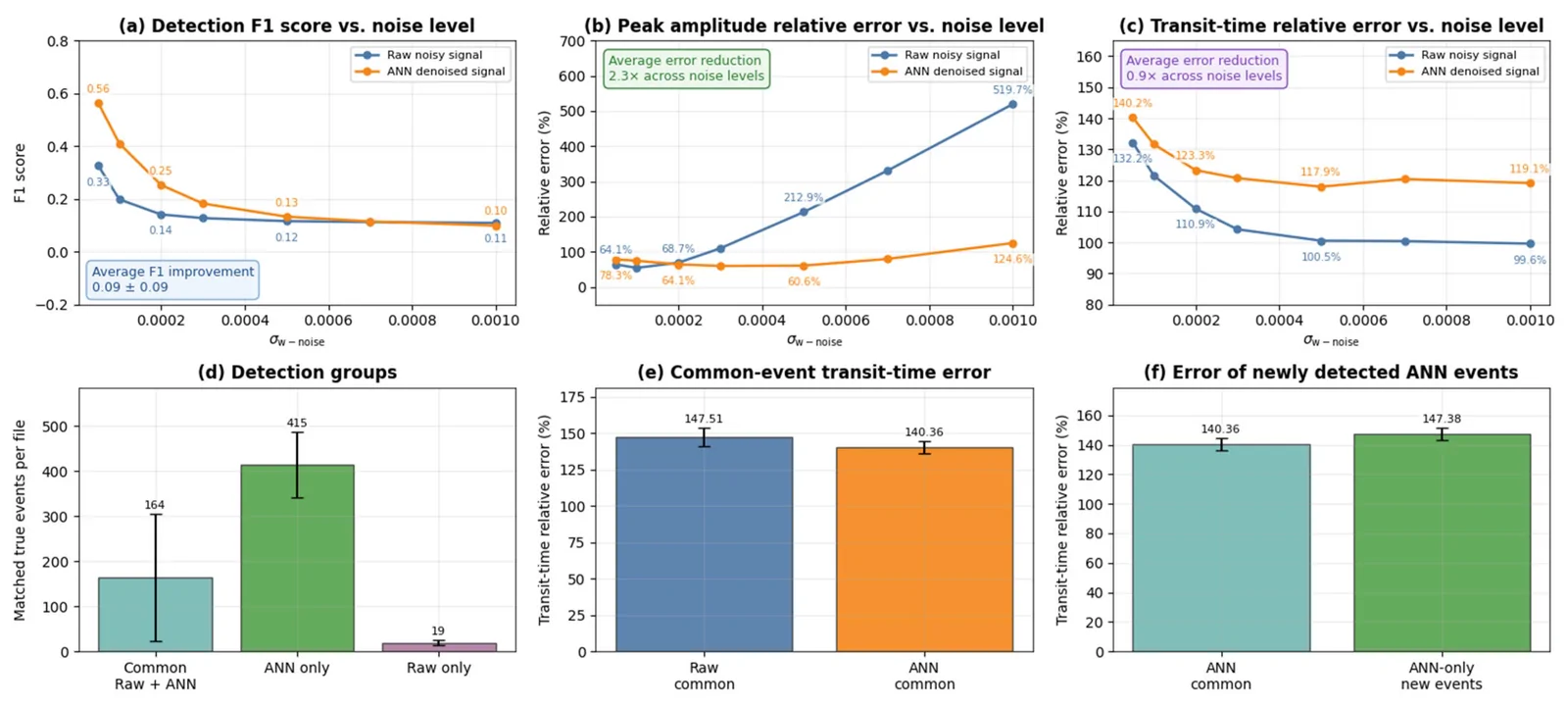
Noise-robustness and detection-set analysis of ANN-denoised synthetic IFC signals. Controlled Gaussian white noise was added to clean synthetic test signals at different standard deviations, $\sigma_{w-noise}$. (**a**–**c**) ANN denoising maintained higher F1 scores across most noise levels and reduced peak-amplitude error, while transit-time error showed only modest improvement. (**d**) Detection-set analysis showed that the ANN recovered many additional true events missed by the raw detector. (**e**) For common events detected by both methods, ANN denoising slightly reduced transit-time error. (**f**) ANN-only events showed slightly higher transit-time error, indicating that newly recovered events were more difficult to characterize.

To test noise robustness, controlled zero-mean Gaussian white noise was added to the clean synthetic test signals over a range of standard deviations, denoted as σ_w-noise_. This design allowed the effect of increasing random noise amplitude to be isolated while keeping the underlying event locations and clean event waveforms fixed. As shown in Figure 6a, both raw and ANN-denoised signals showed decreasing F1 scores as σ_w-noise_ increased, indicating that stronger additive noise progressively reduced event detectability. However, the ANN-denoised signals maintained higher F1 scores than the raw noisy signals across most of the tested noise range. The improvement was most evident at low-to-moderate noise levels, where ANN denoising enhanced weak bipolar event signatures that were partially obscured in the raw signal. At the highest noise levels, the performance gap narrowed, suggesting that severe white noise can limit the recoverable event information even after denoising.

In contrast to the F1 score trend, peak-amplitude estimation showed a more pronounced benefit from ANN denoising. As shown in Figure 6b, the peak-amplitude relative error of the raw signal increased substantially with noise level, whereas the ANN-denoised signal maintained much lower amplitude error across the sweep. On average, the ANN reduced peak-amplitude error by approximately 2.3-fold across the tested noise levels. This result suggests that the denoiser improved the stability of amplitude-related feature extraction by suppressing noise contributions that distort the positive and negative event extrema. Transit-time error showed a different trend from peak-amplitude error (Figure 6c). Unlike amplitude, which is mainly determined by the magnitude of the positive and negative event extrema, transit time depends on the relative positions of these extrema. Therefore, transit-time estimation is highly sensitive to small shifts in peak localization, especially when the event waveform is weak, noisy, or distorted. Although ANN denoising improved event visibility and substantially reduced peak-amplitude error, the average reduction in transit-time error was limited. This result is likely influenced by the larger and more challenging event population recovered after ANN denoising. The raw detector primarily identified clearer, higher-SNR events, whereas the ANN-denoised detector recovered many additional true events that were missed in the raw signal. These additional events are expected to include weaker, more distorted, or less symmetric waveforms, for which the positive and negative extrema are more difficult to localize accurately. As a result, the average transit-time error for the ANN-denoised signal can be affected by the inclusion of these newly detected difficult events.

To examine this detection-set effect, true events were divided into three groups: common events detected by both raw and ANN-denoised signals, ANN-only events detected only after ANN denoising, and raw-only events detected only in the raw signal (Figure 6d). The ANN recovered a large number of additional true events, while the number of raw-only events was small. This confirms that the main contribution of ANN denoising was improved recall through recovery of events that were partially obscured by noise in the raw signal. A more balanced feature comparison was then performed using only the common event population (Figure 6e). For these shared events, “raw common” refers to transit-time error extracted from the raw signal, while “ANN common” refers to transit-time error extracted from the ANN-denoised signal for the same true events. For the common event population, ANN denoising slightly reduced the transit-time error from 147.51% for the raw signal to 140.36% after denoising. This indicates that, for events detected by both methods, ANN denoising did not degrade transit-time estimation and provided a modest improvement in peak-localization consistency. Finally, ANN-only events were compared with ANN common events (Figure 6f). The ANN-only events showed slightly higher transit-time error, supporting the interpretation that the newly recovered events were more difficult to characterize. Therefore, the limited improvement in average transit-time error should not be interpreted as a failure of denoising, but rather as a consequence of detecting a larger and more challenging event population. Together, these results show that ANN denoising improves noise robustness primarily by increasing event detectability and stabilizing amplitude extraction. Transit-time estimation showed a modest improvement for common events, but remained more sensitive to peak-position uncertainty than amplitude estimation. This suggests that the ANN improves the waveform quality for downstream analysis, while further gains in transit-time accuracy may depend on optimization of the feature-extraction step.

### 3.4. Experimental Validation on Real IFC Data

**Figure 7:**
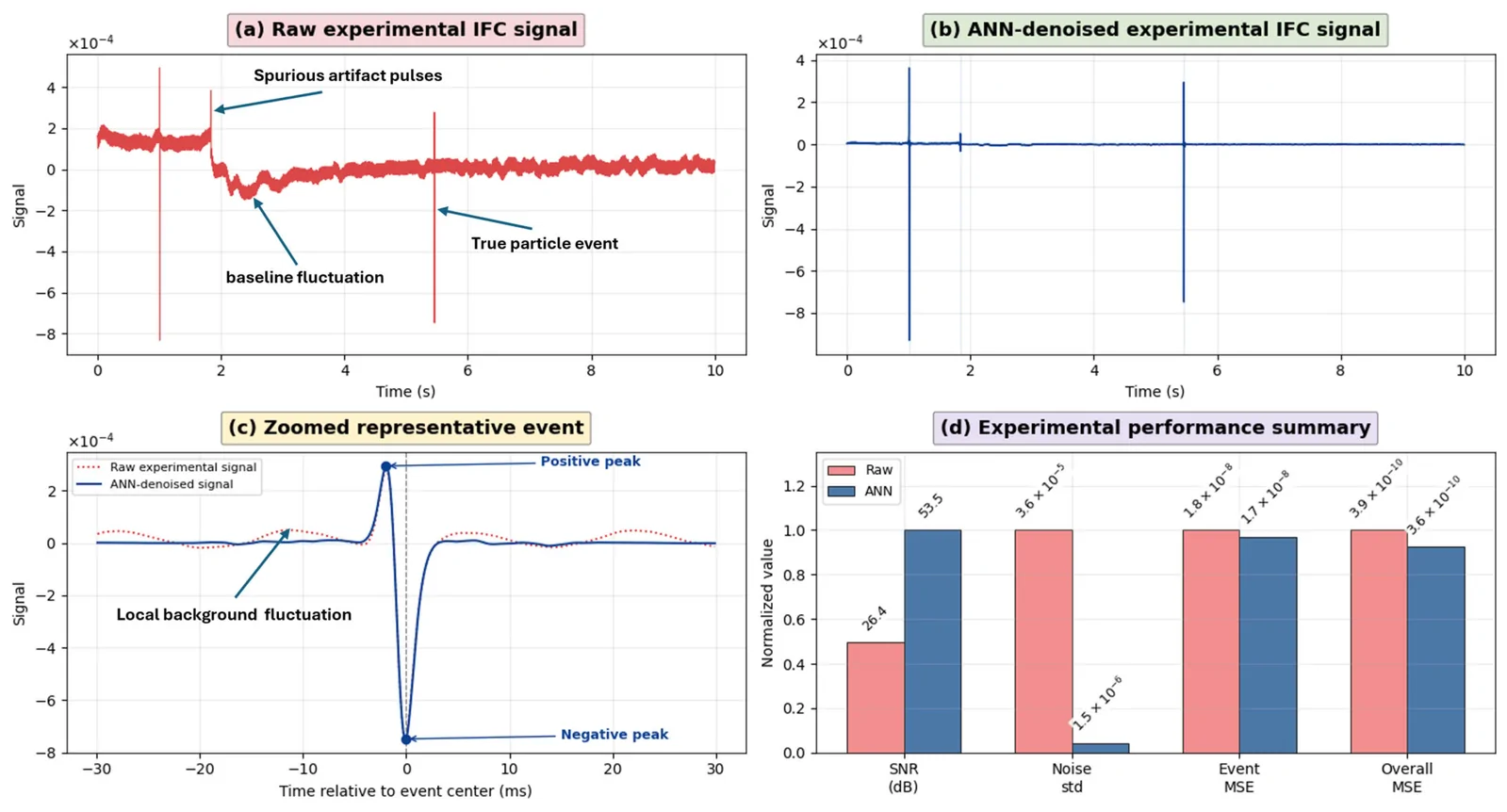
Experimental validation of ANN denoising on real IFC data. (**a**) Raw experimental IFC signal showing baseline fluctuation, spurious artifact pulses, and true particle-event transients. (**b**) ANN-denoised experimental signal with reduced baseline noise and clearer event signatures. (**c**) Zoomed representative event illustrating suppression of local background fluctuations while preserving the bipolar waveform and positive/negative peak structure after denoising. (**d**) Experimental performance summary showing improved SNR and reduced noise standard deviation after ANN denoising, with comparable event-region and overall residual MSE.

To assess whether the model trained exclusively on synthetic signals could generalize to real post-demodulated IFC measurements, the trained ANN was further evaluated using experimentally acquired IFC data. Because experimental IFC recordings can exhibit run-to-run variation in baseline stability, residual interference, flow conditions, and electrode response even under nominally identical acquisition settings, the experimental analysis was used primarily to assess synthetic-to-real transfer and preservation of event morphology rather than particle-size classification performance. This design allows the denoising behavior to be evaluated without introducing additional variability associated with comparisons across independently acquired particle populations. Representative raw and ANN-denoised experimental traces are shown in Figure 7a–c. The raw experimental signal exhibited noticeable baseline fluctuation, background noise, spurious artifact pulses, and true particle-transit events. After ANN denoising, the baseline became substantially more stable, and the particle events were more clearly separated from the background, indicating that the model trained on synthetic paired data was able to generalize to real demodulated IFC measurements and reduce multiple forms of experimental background disturbance. A zoomed representative event shown in Figure 7c illustrates that the denoised signal preserved both the positive and negative extrema associated with particle transit through the sensing region, indicating that the ANN reduced local background fluctuation without eliminating the transient event structure. Despite this overall improvement, the denoised waveform was not completely free of localized residual fluctuations. As shown in Figure 7b, a small localized residual feature remained in the denoised waveform. Its amplitude was considerably lower than those of the dominant particle-transit events in the same recording and is expected to be more readily rejected by the downstream noise-dependent detection threshold. Nevertheless, because the ANN learns from the particle-event waveform and disturbance distributions represented in the synthetic training data, event-like experimental artifacts that resemble trained particle morphology could potentially produce false-positive outputs, while atypical true events outside the training distribution could be attenuated or distorted. This represents an important limitation of the present framework. Future work will therefore include dedicated particle-free control measurements and broader experimental noise conditions to quantify false-positive generation and evaluate robustness to out-of-distribution events.

Experimental summary metrics are shown in Figure 7d. Because no clean ground-truth waveform is available for experimental measurements, the evaluation focused on relative signal-quality indicators rather than absolute reconstruction accuracy. The ANN-denoised signal showed a higher SNR than the raw signal, consistent with the visual reduction in baseline fluctuation and improved event visibility. The estimated noise standard deviation was also strongly reduced after denoising, indicating effective suppression of residual background fluctuations. Event-region residual MSE and overall residual MSE were calculated relative to smoothed versions of the corresponding raw and ANN-denoised signals. These values were used only as relative measures of remaining signal fluctuation, not as ground-truth reconstruction errors. The comparable event-region and overall residual MSE values suggest that ANN denoising did not substantially distort the dominant waveform structure.

Overall, the experimental results support the ability of the ANN trained on synthetic data to generalize to real IFC measurements under a challenging experimental condition. Although the present experimental validation was limited to one particle population and measurement condition, the ANN reduced baseline fluctuation and residual noise while preserving representative bipolar event morphology. Additional validation across different particle sizes, particle-free controls, and broader experimental conditions would further establish the generalizability and robustness of the proposed framework. The proposed framework may have practical value as a software-based enhancement step in IFC systems where weak particle events are partially obscured by residual background fluctuations after demodulation. By improving event visibility while preserving transient waveform morphology, the method could support more reliable particle counting, peak-amplitude estimation, transit-time analysis, and other downstream feature extraction without requiring modification of the sensing hardware. This capability may be particularly relevant to impedance-based biosensing of small particles and micro- to submicron-scale analytes, where transient electrical signals can approach the background-noise level. More broadly, the event-preserving denoising strategy may also be extended to related microfluidic and electrical biosensing platforms that produce sparse transient signals under noisy measurement conditions. Its transferability across different devices, particle or cell types, acquisition settings, and sensing modalities should be evaluated in future studies.

## 4. Conclusions

In this work, we developed an event-preserving artificial neural network framework for post-demodulation signal denoising in impedance flow cytometry. The proposed approach uses paired synthetic IFC signals to train a lightweight multilayer perceptron that maps noisy signal windows to clean event-preserving output windows. By incorporating event-focused sampling and an event-weighted loss function, the model was designed to suppress residual noise and baseline disturbances while preserving the localized bipolar morphology of particle transits.

Synthetic validation demonstrated that the ANN denoiser substantially improved event visibility across simulated 3, 4, and 5 µm beads containing mixed representative noise and artifact components. Compared with raw noisy signals, ANN-denoised signals showed higher recall and F1 score, with the largest benefit observed for smaller, lower-amplitude events. Comparison with representative moving-average and S–G filtering further illustrated the parameter-dependent tradeoff between noise suppression and event preservation in conventional smoothing methods, while the ANN more consistently preserved peak-to-peak event morphology across the representative events. Threshold-sweep analysis further showed that the improvement was maintained across a broad range of detection thresholds and that apparent precision differences could be influenced by threshold selection. Noise-robustness analysis confirmed that ANN denoising improved detection performance and reduced peak-amplitude error under increasing noise levels. Additional detection-set analysis showed that the ANN recovered many true events missed by the raw detector, while maintaining comparable transit-time error for events detected by both methods.

Experimental validation further supported the ability of the model trained on synthetic data to process real IFC measurements under noisy conditions. ANN denoising reduced baseline fluctuation and residual noises in experimentally acquired IFC waveforms while preserving representative bipolar event morphology. Experimental summary metrics showed improved signal-to-noise ratio and reduced estimated noise standard deviation. However, the present validation was limited to one particle population and measurement condition, and broader testing using particle-free controls, additional particle populations, and diverse noise conditions will be needed to further establish robustness and generalizability.

Overall, this study demonstrates that lightweight neural denoising can serve as a practical optional post-demodulation enhancement step for noisy IFC signals, particularly when weak particle events are partially obscured by residual background fluctuations. The proposed framework improves event detectability, supports more stable feature extraction, and provides a practical strategy for IFC signal enhancement without requiring experimentally measured clean reference signals for training, and may provide a useful signal-processing approach for high-sensitivity micro- and submicron-scale impedance sensing.

## Data Availability

The original contributions presented in this study are included in the article. Further inquiries can be directed to the corresponding author.

## Abbreviations

The following abbreviations are used in this manuscript:

IFC	Impedance flow cytometry
ANN	Artificial neural network
MLP	Multilayer perceptron
S–G	Savitzky–Golay
